# Mobile applications in gastrointestinal surgery: a systematic review

**DOI:** 10.1007/s00464-023-10007-y

**Published:** 2023-04-04

**Authors:** Sebastiaan L. van der Storm, Mustafa Bektaş, Esther Z. Barsom, Marlies P. Schijven

**Affiliations:** 1 Amsterdam UMC Location University of Amsterdam, Surgery, Meibergdreef 9, Amsterdam, The Netherlands; 2 Amsterdam Gastroenterology and Metabolism, Amsterdam, The Netherlands; 3 Amsterdam Public Health, Digital Health, Box 22660, 1105 AZ Amsterdam, The Netherlands

**Keywords:** Mobile applications, Apps, Gastrointestinal surgery, mHealth, Digital health, Mobile healthcare

## Abstract

**Background:**

Mobile applications can facilitate or improve gastrointestinal surgical care by benefiting patients, healthcare providers, or both. The extent to which applications are currently in use in gastrointestinal surgical care is largely unknown, as reported in literature. This systematic review was conducted to provide an overview of the available gastrointestinal surgical applications and evaluate their prospects for surgical care provision.

**Methods:**

The PubMed, EMBASE and Cochrane databases were searched for articles up to October 6th 2022. Articles were considered eligible if they assessed or described mobile applications used in a gastrointestinal surgery setting for healthcare purposes. Two authors independently evaluated selected studies and extracted data for analysis. Descriptive data analysis was conducted. The revised Cochrane risk of bias (RoB-2) tool and ROBINS-I assessment tool were used to determine the methodological quality of studies.

**Results:**

Thirty-eight articles describing twenty-nine applications were included. The applications were classified into seven categories: monitoring, weight loss, postoperative recovery, education, communication, prognosis, and clinical decision-making. Most applications were reported for colorectal surgery, half of which focused on monitoring. Overall, a low-quality evidence was found. Most applications have only been evaluated on their usability or feasibility but not on the proposed clinical benefits. Studies with high quality evidence were identified in the areas of colorectal (2), hepatopancreatobiliary (1) and bariatric surgery (1), reporting significantly positive outcomes in terms of postoperative recovery, complications and weight loss.

**Conclusions:**

The interest for applications and their use in gastrointestinal surgery is increasing. From our study, it appears that most studies using applications fail to report adequate clinical evaluation, and do not provide evidence on the effectiveness or safety of applications. Clinical evaluation of objective outcomes is much needed to evaluate the efficacy, quality and safety of applications being used as a medical device across user groups and settings.

**Supplementary Information:**

The online version contains supplementary material available at 10.1007/s00464-023-10007-y.

The use of smartphones and mobile application software (apps) is deeply integrated into society and their potential is being increasingly recognized in healthcare. In the past decade, the development of healthcare apps has rapidly increased, with the intention of providing medical solutions to some extent. At present, over 400.000 healthcare apps are available for download in mobile app stores worldwide [1].

To date, the number of apps used in gastrointestinal surgical care is limited compared with that in other surgical disciplines [2]. This may change rapidly. Apps are believed to offer great possibilities to support or improve gastrointestinal surgical care, and overall healthcare is on the lookout of the smart use of digital solutions in times of limited resources. Apps may facilitate patients, healthcare providers (HCP), or both. Apps have the potential to improve information provision, communication between patients and HCP, clinical decision-making, perioperative guidance and monitoring, and education/training. In addition, apps may be used to register clinically relevant variables as apps can be developed to connect with sensors or other measurement devices such as a camera, an activity tracker, a biosensor, or a blood pressure monitoring device [3–5].

The use of apps in healthcare is not without controversy or debate [6, 7]. As apps may influence patient-reported or clinical outcomes, they must be properly developed and validated. Apps or software in general to be used as a medical device must comply with standards as described by the European Medical Device Regulation (MDR) or the American Food and Drug Administration (FDA), safeguarding the quality and safety of the app [8, 9]. However, the distribution of apps is limitedly regulated by the app stores, with minimum supervision on whether these specific legislations are indeed met. Even if they are met, it is not guaranteed that the use of the app will lead to valid and reliable results across situations and user settings [7, 10]. For that, scientific research validating apps with well-designed research protocols is required. To date, a clear overview of properly validated gastrointestinal surgical apps is lacking. Therefore, this systematic review focuses on the following research questions: (1) Which apps that are used in gastrointestinal surgical care have been described in literature? (2) Are these apps clinically evaluated on objective outcomes and able to improve gastrointestinal surgical care?

## Methods

This systematic review was conducted in line with the Cochrane Handbook for Systematic Reviews of Interventions version 6.0 and reported according to PRISMA 2020 [11]. This study was registered in Open Science Framework (https://doi.org/10.17605/OSF.IO/X56RA. Studies were considered eligible if they assessed or described mobile apps used in a gastrointestinal surgery setting and were published in 2010 or later. The search was last updated October 6th 2022. A mobile app is defined as a software program which operates only on a smartphone or tablet (and thus, not web-based software). Keywords related to mobile apps and gastrointestinal surgery were incorporated into the search strategy. The search string is presented in the appendix. The included articles were cross-referenced to identify any additional relevant studies. Studies were excluded if (1) the described mobile app was only used to register study outcomes (e.g. number of complications and operation time), (2) the articles were conference proceedings or study abstracts, as they do not provide adequate insights into the app or its evaluation, (3) reviews, and (4) the results were published in a language other than English. Two reviewers (SvdS and MB) independently assessed all titles and abstracts according to the inclusion and exclusion criteria in the software tool “Rayyan”. Studies were included in the full-text evaluation when both reviewers agreed on inclusion. Disagreements were resolved through appraisal by a third reviewer (EB).

The methodological quality of the randomized controlled trials was assessed using the Revised Cochrane risk of bias tool for randomized trials (RoB-2) [12]. This tool determines the overall risk of bias that is based on the randomization process, deviations from intended interventions, missing outcome data, measurement of outcomes and selection of reported results. The ROBINS-I tool was used to determine the methodological quality of non-randomized studies, in which the overall risk of bias is based confounding, participant selection, intervention classification, deviations from intended interventions, missing outcome data, measurement of outcomes, and selection of reported results [13].

Data were extracted independently by two reviewers (SvdS and MB) in a standardized form that included: year of publication, country, study design, number of participants, characteristics of included participants, type of surgery, name of the app, platform of the app, functionalities of the app, and study outcomes. All study outcomes on usability, satisfaction and clinical outcomes were included because apps may have heterogeneous aims and functionalities. Conflicts among reviewers were resolved by consensus. The results of studies were summarized according to the apps described. The apps were categorized based on their functionalities to provide a structured overview of available apps. The apps were described within these categories and were assessed on their outcome evaluations.

## Results

**Fig. 1:**
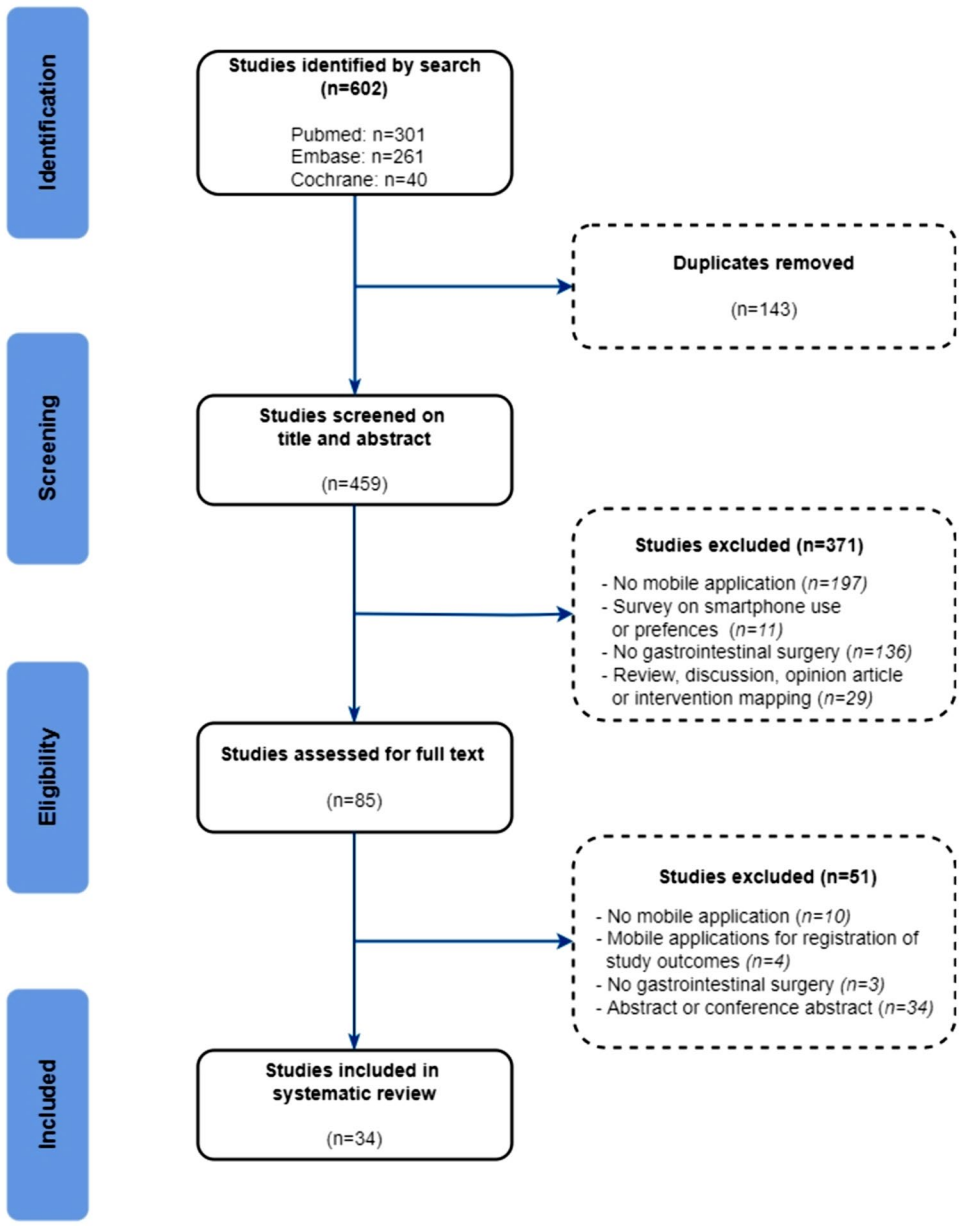
The PRISMA flow diagram

**Fig. 2:**
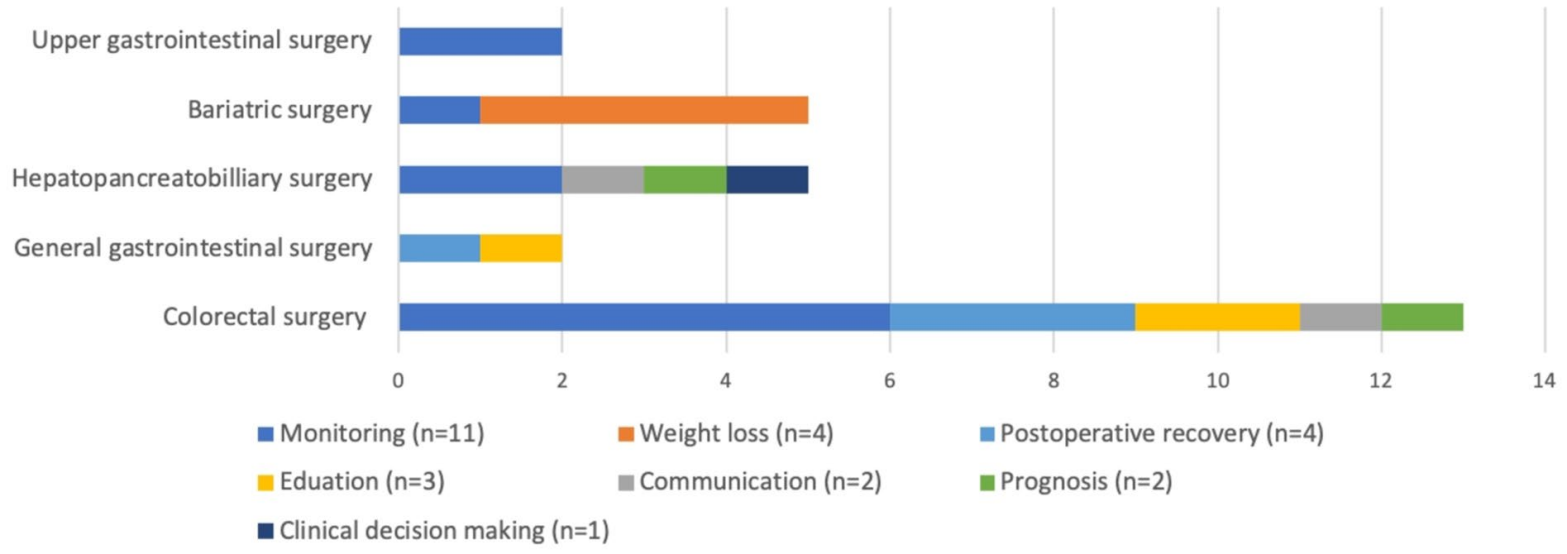
Seven categories of apps in the gastrointestinal surgical domain (*N* = 29)

In total, 477 studies were screened for eligibility based on their title and abstract. After a full-text assessment, 38 studies were included of which 29 apps were described (Fig. 1). Patients were targeted as users in all apps except in three apps which were used by surgeons [45, 48, 53]. The apps were classified into seven categories: monitoring, weight loss, postoperative recovery, education, communication, prognosis, and clinical decision-making. The majority of the studies focused on colorectal surgery and monitoring (Fig. 2). An overview of the study’s characteristics is presented in Table 1. Due to the heterogeneity of the study designs and apps, a meta-analysis was impeded. In total, seven randomized control trials and seven comparative cohort studies were included. Only four studies had an overall low risk of bias as summarized in Tables 2, 3 [33, 38, 42, 53].

**Table 1:** General characteristics of included studies

Study	Country	Study design	Control group	Follow-up	Surgical procedure	Participants			App category	Main app functions	System	Study outcomes
						Type	*N* =	Age (mean)				
Keng 2020^a^	Canada	Cross-sectional	No	30 days	Colorectal surgery	Patients	82	43	Monitoring	- Self-reported assessment on symptoms - Informative library - Photograph function	iOS & Android	- Overall completion of daily assessments 41–64% - 92% patients with a good overall satisfaction (26% completed the questionnaire) - 30-day readmission rate of 6%
Pooni 2022^a^	Canada	RCT	Yes	30 days	Colorectal surgery	Patients	128; 125	41; 50	Monitoring	- Self-reported assessment on symptoms - Informative library - Photograph function	iOS & Android	- No difference in postoperative outcomes - Improved patient-reported outcomes (satisfaction, well-being & anxiety
Anpalagan 2022^a^	Canada	Study protocol RCT	Yes	30 days	Colorectal surgery	Patients	670	–	Monitoring	- Self-reported assessment on symptoms - Informative library - Photograph function	iOS & Android	- Unplanned hospital visits within 30 days - Quality of life
Lee 2021^a^	Canada	Prospective cross-sectional survey	Yes historical	30 days	Colorectal surgery	Patients	48; 73	60; 57	Monitoring	- Self-reported assessment on symptoms - Education material - Photograph function - Chat functionality with HCP’s	iOS & Android	- Completion of a daily assessment at least once 57% - 80% patients with a good overall satisfaction - Similar postoperative outcomes with control group
Lee 2022^a^	Canada	Prospective Cohort	Yes	30 days	Colorectal Surgery	Patients	70; 35	59; 55	Monitoring	- Self-reported assessment on symptoms - Education material - Photograph function - Chat functionality with HCP’s	iOS & Android	- Similar postoperative outcomes with control group
Eustache 2021^a^	Canada	Prospective cohort study compared to retrospective cohort	Yes	30 day	Colorectal surgery	Patients	94; 256	55; 56	Monitoring	- Self-reported assessment on symptoms - Education material - Photograph function - Chat functionality with HCP’s	iOS & Android	- Usability score of 84.5 (0–100) - Significant decrease in potentially preventable 30-day emergency visits (incidence rate 0.34) - Significant decrease in length of stay (3.2 vs 4.6 days) - No difference other postoperative outcomes
Agri 2020	Switzerland	Retrospective Cohort	No	30 days	Colorectal surgery	Patients	43	54	Monitoring	- Self-reported assessment on symptoms - Informative library - Alert messages which was send to HCP’s	iOS & Android	- Overall completion of daily assessments of 72% - 4/5 level of patient satisfactio (30% completed the questionnaire) - All postoperative outcomes were detected - Median response time of 90 min of the HCP
Symer 2017	USA	Pilot Study	No	30 days	Colorectal surgery	Patients	21	52	Monitoring	- Self-reported assessment on symptoms - Alert messages - Photograph function - Connection with activity tracker	iOS & Android	- 84% patients completed at least 70% daily task - 2,7/5 level of patient satisfaction - 26,7%patients received alerts based on symptom assessments - Mean return to baseline activity of 30 days
Diehl 2021^b^	US	Pilot Study	No	30 days	- Colorectal surgery (68%) - Oncological surgery (32%)	Patients	50	50	Monitoring	- Education materials - Notifications - Self-reported assessment on symptoms reviewed by HCP’s	iOS & Android	- Engagement with individual app features 48–81%
Diehl 2021^b^	US	Study protocol RCT	Yes	180 days	- Colorectal surgery - Oncological surgery - Transplant surgery	Patients	300 (sample size)	–	Monitoring	- Education materials - Notifications - Self-reported assessment on symptoms reviewed by HCP’s	iOS & Android	- Hospital readmission - Urgent care visits - Complications - Total readmission costs
Valk 2022	Canada	Study protocol Feasibility RCT	Yes	42 days	Colorectal surgery	Patients	80 (sample size)	–	Monitoring	- Self-reported assessment on symptoms - Photograph function	iOS & Android	- Usability / app engagement
Gustavell 2019^c^	Sweden	Pilot Study	No	30 days	Hepato-pancreatobiliary surgery	Patients	6	65	Monitoring	- Risk assessment model for alerts - Self-reported assessment on symptoms - Graph of symptoms	iOS & Android	- Overall completion of daily assessments 84% - Patient’s experiences
Gustavell 2019* ^c^	Sweden	Cohort	Yes historical	6 months	Hepato-pancreatobiliary surgery	Patients	26; 33	67; 66	Monitoring	- Self-reported assessment for symptoms - Risk assessment model for alerts - Graph of symptoms	iOS & Android	- Overall completion of assessments 83-95% - Significantly less reported hepatic symptoms and higher self-care
2020	Sweden	Cohort	No	6 months	Hepato-pancreatobiliary surgery	Patients	26	67	Monitoring		iOS & Android	- Patient’s experiences
Allenson 2021	US	Pilot study	No	30 days	Hepato-pancreatobiliary surgery	Patients	19	65	Monitoring	- Self-reported assessment of dietary intake - Nutrition goals	NS	- 79% patients completed at least 80% daily task - 89% patients with a good overall satisfaction - Average of 82,4% caloric goals intake
Wu 2019	Taiwan	Feasibility study	No	28 days	Upper Gastrointestinal surgery	Patients	43	68	Monitoring	- Education materials - Monitoring of symptoms, body weight, physical activity - Photograph function	iOS & Android	- Overall completion of assessments 96%
Chlan 2021	US	Mixed methods	No	1 year	Upper Gastrointestinal surgery	Patients	50	63	Monitoring	- Self-reported assessment on symptoms - Graph of symptoms	NS	- 98% patients reached 90% feasibility threshold - Patient’s experiences
Heuser 2021	Canada	Retrospective Cohort	Yes	30 days	Bariatric surgery	Patients	396; 458	45; 48	Monitoring	- Informative library - Daily recovery milestones - Daily questionnaires	iOS & Android	- Completion of daily assessments at least once a week 66% - 90% patients with a good overall satisfaction - 49% patients reported that the app helped to avoid phone calls - No improvement on postoperative outcomes
Mangieri 2019	US	RCT	Yes	24 months	Bariatric surgery	Patients	28; 28	53; 53	Weight loss	- Nutritional information - Self-reported assessment on intake and weight - Personalized diet program	iOS & Android	- Significant more weight loss after 1 year (81.4% vs 74.4%) - Significant more weight loss after 2 years (71.5% vs 59.1%) - No difference in quality of life
Dolan 2019	US	Prospective Cohort	No	30 days	Bariatric surgery	Patients	10	38	Weight loss	- Self-reported assessment on intake and symptoms - Informative library - Push notifications - Activity tracker	NS	- 84% patients completed at least 70% of daily task - 2,7/5 level of patient satisfaction
Sysko 2022	US	Pilot RCT	Yes	8 weeks	Bariatric surgery	Patients	25; 25	40; 38	Weight loss	- Informative library - Self-reported assessment on intake and weight - Social challenges and feedback - Activity tracker	NS	- Effect size stress -0.58 - Effect size anxiety -0.62 - No difference in the caloric intake, weight loss or quality of life
Mundi 2015	US	Feasibility study	No	4 months	Bariatric surgery	Patients	30	41	Weight loss	- Automatic text messages	iOS & Android	- 31% response rate - 7.3 kg weight loss
Bonn 2020	Sweden	Study Protocol RCT	Yes	24 months	Bariatric surgery	Patients	154 (sample size)	-	Weight loss	- Informative library - Daily milestones on activity and vitamin intake - Feedback on activity and vitamin intake - Daily questionnaires - Tracking activity using an accelerometer	iOS & Android	- Level of physical activity - Weight loss
Van der Meij 2018^d^	Netherlands	RCT	Yes	6 months	- General gastrointestinal surgery - Gynecologic surgery	Patients	171; 173	52; 51	Post-operative recovery	- Informative library - Feedback on the postoperative recovery process - Connection with activity tracker - E-consult direct contact with HCP’s	iOS & Android	- Significant decrease of time to return to daily activities (21 vs 26 days) - No difference in postoperative outcomes - Improved satisfaction with care program (7.2 vs 6.3)
Den Bakker 2019^d^	Netherlands	Mixed methods process	No	3 months	General gastrointestinal surgery	Patients	73	63	Post-operative recovery	- Informative library - Feedback on the postoperative recovery process	iOS & Android	- App engagement 63%, activity tracker engagement 67% - Patient satisfaction with the app 7,5/10
		Semi-structured interview					14	62		- Connection with activity tracker - E-consult direct contact with HCP’s		- Patients’ barriers and facilitators for use of the intervention
Pecorelli 2017^e^	Canada	Pilot Study	No	28 days	Colorectal surgery	Patients	45	61	Post-operative recovery	- Informative library - Feedback on the postoperative recovery process - Daily recovery milestones - Daily questionnaires	NS	- Usability score of 85 (0–100) - 89% patients with a good overall satisfaction
Mata 2019^e^	Canada	RCT	Yes	30 days	Colorectal surgery	Patients	50; 47	63; 57	Post-operative recovery	- Informative library - Feedback on the postoperative recovery process - Daily recovery milestones - Daily questionnaires	iPad (iOS)	- Non-significant difference in protocol adherence (59% vs 62%) - No difference in postoperative outcomes
Rauwerdink 2019	Netherlands	Study Protocol RCT	Yes	42 days	Colorectal surgery	Patients	156 (sample size)	–	Post-operative recovery	- Informative library - Daily recovery milestones - Push notifications - Daily questionnaires - Connection with activity tracker	iOS & Android	- Adherence to recovery protocol - Postoperative outcomes - Satisfaction
Bertocchi 2021	Italy	Study protocol observational study	No	-	Colorectal surgery	Patients	270 (sample size)	–	Post-operative recovery	- Education materials - Daily recovery milestones - Push notifications - Self-reported assessment for symptoms	iOS & Android	- Confidence using the app - Compliance ERAS elements - Hospital stay, admission rate, complications
Kowalewski 2017	Germany	Validation study	Yes	–	General gastrointestinal surgery	Surgeons, residents, students	54; 51	NS	Education	- Cognitive task simulation - Practice of surgical procedures	iOS	- Surgeons significantly outperformed students (construct validity) - The app aids in the learning and assessment process of the necessary aspects (content validity) - The app represents the reality of the training situation (face validity)
Gaj 2017	Italy	RCT	Yes	–	Colorectal surgery	Patients	63; 63	35; 32	Education	- 3D model of lower abdomen	NS	- Significantly higher degree of clarity doctor (4.4 vs 3.5) - Significantly higher patient satisfaction (4.2 vs 3.5)
Yiğitoğlu 2021	Turkey	Prospective cohort	Yes	3 months	Colorectal (ostomy)	Patients	30 60	51; 55	Education	Education materials	Android	- No difference in psychosocial adjustment - No difference in stoma-related problems
Nardo 2016	Italy	Cohort	Yes	28 months	Hepato-pancreatobiliary surgery	Patients^#^	19; 27	63; 64	Communication	- Text messages - Sending image, or other files	iOS & Android	- Averagely 32 communication events a month: clinical questions (54%), instructive comments (32%), administration questions (14%) - No differences in postoperative outcomes
Doğan 2022	Turkey	RCT	Yes	3 months	Bariatric surgery	Patients	26; 25	36.5 39.9	Communication	- Live consultation - Informative library - Nutrition and activity diary	Android	- Significant difference in BMI postoperative - No difference in other postoperative outcomes
Moon 2021	Canada	Study protocol RCT	Yes	6 months	Colorectal Surgery	Patients	462 (sample size)	-	Communication	-Online informative modules -Peer support platform	iOS & Android	- Quality of life - Patient activation - Bowel function
Gabriel 2015	US	Retrospective Cohort	No	-	Colorectal surgery	Patients	34.176	69	Prognosis	- Survival rate calculator	iOS & Android	- Development of the app (no evaluation of the app)
Low 2021		Prospective longitudinal study	No	60 days	Hepato-pancreatobiliary surgery	Patients	44	66	Prognosis	- Self-reported assessment for symptoms - Collection of smartphone data (location, movement, device use, noise and light levels)	Android	- 73.5% accuracy of the prediction of symptoms during the next day - No evaluation of the app
Smits 2022	Netherlands	RCT	Yes		Hepato-pancreatobiliary surgery	Patients^#^	863; 885	66; 65	Diagnostic and therapeutic decision-making	- Algorithm based on clinical and biochemical variables	iOS & Android	- 94% daily data entry - 81% overall adherence to algorithm - Significant reduction of postoperative complications: bleeding (5% vs 6%), organ failure (5% vs 10%) and 90-day mortality (3% vs %)

*Multiple studies using the same database

#The app was used by surgeons

a, b, c, d, eStudies evaluating the same mobile application

*RCT* randomized controlled trial, *NA* not applicable, *NS* Not specified

**Table 2:** An overview of the methodological quality assessment of the RCTs according to the Revised Cochrane risk-of-bias tool for randomized trials

Studies	Bias in randomization process	Deviations from intended interventions	Missing outcome data	Bias in outcome measurements	Bias in reported results	Overall risk of bias
Pooni 2022	High	Some concerns	Low	Low	Low	High
Anpalagan 2022*	Low	Low	NA	NA	NA	NA
Diehl 2022 *	Low	Low	NA	NA	NA	NA
Valk 2022*	Some concerns	Low	NA	NA	NA	NA
Mangieri 2019	Low	Low	Low	Low	Low	Low
Sysko 2022	Low	Low	Low	Low	Some concerns	Some concerns
Bonn 2020*	Low	Low	NA	NA	NA	NA
Van der Meij 2018	Low	Low	Low	Low	Low	Low
Mata 2020	Low	Low	Low	Low	Low	Low
Rauwerdink 2019*	Low	Low	NA	NA	NA	NA
Doğan 2022	Some concerns	Some concerns	Low	Some concerns	Low	Some concerns
Moon 2021*	Low	Low	NA	NA	NA	NA
Gaj 2017	Low	Low	Low	Some concerns	Low	Some concerns
Smits 2022	Low	Low	Low	Low	Low	Low

*Study protocols for which the methodological quality could not be fully assessed

*NA* not applicable

**Table 3:** An overview of the methodological quality assessment of the non-randomized studies according to the ROBINS-I assessment tool

Studies	Bias due to confounding	Bias in participant selection	Bias in intervention classification	Bias due to deviations from intended interventions	Missing data	Bias in outcomes measurements	Bias in reported results	Overall risk of bias
Keng 2016	Moderate	Moderate	Low	Low	Serious	Moderate	Moderate	Serious
Lee 2021	Serious	Low	Moderate	Serious	Serious	Moderate	Moderate	Serious
Lee 2022	Moderate	Low	Moderate	Moderate	Moderate	Serious	Moderate	Serious
Eustache 2021	Low	Low	Low	Moderate	Moderate	Moderate	Low	Moderate
Agri 2020	Moderate	Moderate	Moderate	Low	Moderate	Moderate	Moderate	Moderate
Symer 2017	Moderate	Moderate	Moderate	Moderate	Moderate	Moderate	Moderate	Moderate
Diehl 2021	Serious	Moderate	Moderate	Low	Low	Moderate	Moderate	Serious
Gustavell 2019	Serious	Moderate	Low	Moderate	Low	Serious	Serious	Serious
Gustavell 2020*	Moderate	Moderate	Low	Low	Low	Moderate	Serious	Serious
Gustavell 2019*	Moderate	Low	Low	Low	Low	Low	Moderate	Moderate
Allenson 2021	Low	Moderate	Moderate	Low	Low	Moderate	Low	Moderate
Wu 2019	Moderate	Moderate	Low	Moderate	Low	Moderate	Serious	Serious
Chlan 2022	Moderate	Low	Serious	Moderate	Moderate	Serious	Moderate	Serious
Heuser 2021	Moderate	Moderate	Moderate	Moderate	Low	Moderate	Low	Moderate
Dolan 2019	Serious	Serious	Serious	Serious	Moderate	Moderate	Low	Serious
Mundi 2015	Moderate	Moderate	Low	Moderate	Low	Serious	Serious	Serious
Den Bakker 2019	Moderate	Serious	Low	Moderate	Moderate	Serious	Low	Serious
Pecorelli 2018	Moderate	Moderate	Low	Low	Moderate	Moderate	Low	Moderate
Berthocchi 2020	Moderate	Moderate	Moderate	NA	NA	NA	NA	NA
Kowalewski 2017	Moderate	Moderate	Low	Low	Low	Moderate	Moderate	Moderate
Yiğitoğlu 2021	Serious	Moderate	Moderate	Moderate	Moderate	Serious	Moderate	Serious
Nardo 2016	Serious	Moderate	Moderate	Moderate	Moderate	Serious	Moderate	Serious
Gabriel 2016	Low	Low	NA	NA	Low	Low	Low	NA
Low 2022	Moderate	Low	Moderate	Moderate	Moderate	Serious	Moderate	Serious

*Multiple studies within the same database

*NA* not applicable

### Monitoring

Almost half of the identified apps were used to monitor the clinical condition of patients who underwent gastrointestinal surgery [14–34]. In general, the monitoring apps provided information about the operation, postoperative care, and self-management, contained daily assessments of the surgical wound (image uploading), symptoms and recovery progress, and some apps shared this information with the HCP.

Six apps monitored patients after colorectal surgery. These apps had a completion rate of the daily assessments between 21 and 84%, and had good patient satisfaction. [14–24]. The app of Keng et al. had a 30-day readmission rate of 6% in comparison with a reported rate of 18% prior to the start of the cohort study [14]. However, postoperative outcomes were not improved in a randomized controlled trial (RCT); only patient-reported outcomes did improve [15]. In another RCT, it will be evaluated whether the app could prevent unplanned hospital visits [16]. The app “Caresense” also had a communication feature. The app was evaluated in combination with the same-day discharge (SDD) protocol. The postoperative outcomes of patients using the app were comparable to patient without the app[17, 18]. The app was also evaluated in a retrospective study, in which the patient did not follow the SSD protocol. The app significantly decreased the rate of preventable emergency department visits [19]. The app is available in the app stores, but not freely accessible. The app “Maela” was successfully tested on it feasibility and all post-discharge complications were detected by the app [20]. The app is available in the app stores, but not freely accessible. The app of Symer et al. generated alerts for 26,7% of the patients and one patient within this group was readmitted [21]. The app “MobiMD” was initially developed for several gastrointestinal procedures but its feasibility was successfully tested on mainly colorectal patients [22]. The effect of the app on hospital readmissions will be evaluated in a RCT [23]. The app “how2trak” is focused on surgical wound and symptom surveillance and its feasibility evaluation has not yet been completed [24].

Two apps monitored patients after undergoing hepatopancreatobiliary surgery and both had a high reporting adherence [25–28]. The “Interaktor” app was evaluated in a cohort, in which patients using the app reported significantly less symptoms and higher self-care activity rates compared to a historical control group[25–27]. The app is available in the app stores. The already available “MyPlate” app monitored postoperative dietary intake and was used by the dietitian to guide patients during counseling visits. Caloric goals were achieved by 82.4% of the patients [28].

Two apps monitored patients after upper gastrointestinal surgery and both were globally tested on their feasibility [29–31]. The app “SurgeryDiary” had a high overall daily submission rate [29]. The app “UDD” (Upper Digestive Disease) was indicated as a helpful tool for reporting and identifying problems, and enhanced communication with HCP [30]. However, the scoring of dumping-related symptoms and pain which was used in the app was not yet adequate [31].

One app monitored bariatric patients and provided advice on whether the patients were on track or to seek symptom management by reviewing the educational materials or contacting a HCP [32]. The app was evaluated in a cohort in which clinical outcomes such as hospital stay or readmission did not differ between app users and the control group. Although adherence was relatively low, most patients were satisfied with the app.

### Weight loss

Two apps mainly focused on a healthy diet, provided nutritional information and allowed bariatric patients to monitor their intake and weight [33, 34]. The already available app “MyfitnessPal” also allowed patients to make a diet program. The app was clinically evaluated in a RCT in which the control group was not allowed to use the app and only received self-monitoring journals [33]. The percentage of weight loss after two years was significantly higher for patients using the app (71,5%) than for those who did not use the app (59,1%). The other app, developed by Dolan et al., had high adherence, but a relatively low patient satisfaction [34].

The other three apps were aimed at engagement and stimulation of physical activity and a healthy diet of bariatric patients [35–37]. The extensive app of Sysko et al. was provided in combination with eight weekly virtual check-ins to review weight loss and the overall process before bariatric surgery [35]. The app was evaluated in a pilot RCT. On average, patients opened the app five times per week and entered their weight twice per week. Patients using the app showed a significant moderate decrease in stress and anxiety, whereas the effect on the caloric intake, weight loss and quality of life did not improve. The app of Mundi et al. provided automatic text messages stimulating a healthy lifestyle, and patients using this app had an average postoperative weight loss of 7.3 kg [36]. The app “PromMera” monitors and stimulates physical activity and self-registered vitamin intake, but its clinical evaluation in a RCT has not yet been completed [37].

### Postoperative recovery

Four apps intended to improve postoperative recovery, providing perioperative information and feedback on the postoperative recovery process [34–40]. The app “IkHerstel” (I recover) was initially developed for gynecological patients and adapted to fit a general gastrointestinal surgical population [38]. The app was evaluated in a RCT, in which the control group received access to a placebo website containing standard general information [39]. The time until postoperative return to normal daily activities significantly was shortened of four days in the intervention group (21 vs 25 days), whereas other postoperative complications did not differ. Patients were satisfied with the app and had relatively high involvement with the app and the activity tracker [40]. The app is available in the app stores, but not freely accessible.

The other three apps were more focused on improving compliance to the recovery protocol after colorectal surgery, providing daily recovery milestones, and questionnaires to track patient compliance and assess patient-reported outcomes [37–40]. The app of Pecorelli et al. had a high usability score and patient satisfaction [41]. Subsequently, the app was evaluated in a RCT in which overall adherence to the postoperative recovery protocol and other postoperative outcomes did not improve [42]. The app “ERAS APPtimisation” specifically targets patient related elements of the Enhanced Recovery After Surgery (ERAS) protocol, and daily activity was monitored and simulated using an activity tracker [43]. The clinical evaluation in a RCT has not yet been completed. The comparable “IColon” app which incorporated slightly different ERAS elements, will be clinically evaluated in an observational study [44].

### Educational apps

The “Touch Surgery” app facilitated three modules for laparoscopy to practice surgical procedures and cognitive tasks. Although the app was successfully validated based on its construct, face and content, training with the app did not improve students’ performance on a VR trainer [45]. The app is freely available in the app stores.

The app “Iprocto” provided a 3D model of various structures in the lower abdomen to improve the information provision to patients during the preoperative consult [46]. The intervention group used this app during consultations, whereas the control group did not use the app. The intervention group reported significantly higher scores of the clarity on the doctor and satisfaction regarding the proctologic visit than the control group.

The “Stoma-M” app provided educational information and contact details of stoma care units and associations in Turkey [47]. The app was evaluated in a quasi-experimental study, in which the intervention group received the app on a provided Android phone, while the control group received a booklet containing the same content as provided in the app. The app did not improve psychosocial adaptation and stoma-related problems.

### Communication

The commonly known app “WhatsApp” was evaluated as a communication tool among surgeons [48]. In this study, surgeons treated patients in two cohorts:1) surgeons who communicated using traditional procedures, such as e-mail, phone calls, and collegial meetings, or 2) surgeons who used the “WhatsApp Surgery Group”, in which surgeons could communicate with each other. No differences in surgical clinical outcomes were reported between the two groups.

The app of Doğan et al. enabled bariatric patients to have a live consultation with researchers and contained educational materials [49]. The app did not improve self-care, quality of life and the self-body image. Although significant differences in BMI were reported between the intervention and the control group, the weight loss towards the preoperative weight was not analyzed.

Moon et al. developed a peer support app for patients with low anterior resection syndrome [50]. The app consisted of information modules and a peer support forum in which patients could communicate with mentors monitored by a team of HCP’s. The app will be evaluated in a RCT on its impact on patients-reported outcomes.

### Prognosis

The app of Gabriel et al. contained a prediction model of the 5 years overall survival of postoperative patients with stage II or III colon cancer which was based on a large retrospective cohort study [51]. However, the app itself has not been tested on its usability, effectiveness and reliability in clinical care.

The already available “AWARE” app collected behavioral data of patients after pancreatic surgery, which was used in combination with an activity tracker to predict postoperative symptoms with a 73.5% accuracy [52]. However, the prediction was calculated afterwards and was not included in the app. Thus, the clinical relevance of the app has not been evaluated.

### Clinical decision-making

The app “Pancreatic Surgery” contained a multimodal algorithm for early recognition and minimally invasive management of postoperative complications after pancreatic surgery, in which the HCP were instructed to enter data daily. The app was evaluated in a RTC, and patients who were treated in accordance with the algorithm in the app had significantly less postoperative complications than those who received usual care [53]. The app is freely available in the app stores.

## Discussion

Healthcare apps may offer great possibilities to support or improve gastrointestinal surgical care, provided that the development and validation process are properly conducted and the app itself complies with professional standards and medical device regulations [8, 9]. This systematic review showed that most the gastrointestinal apps, which have been described in literature, at best had a low-quality evidence and were limited in their evaluation methodology. Small sample sizes, lack of comparison with a control group and subjective outcomes defined were common limitations. Most of the identified apps were only assessed on their usage, usability, satisfaction and feasibility, which was rarely measured with a valid and reusable questionnaire. Studies of higher-level evidence in the area of colorectal [38, 42]. Hepatopancreatobiliary [53] and bariatric surgery [33] reported mostly positive outcomes on postoperative recovery, complications and weight loss.

In total, the review retrieved 29 apps developed for use by patients, surgeons, or both. In the selected studies, there was a predominant focus on monitoring the patient’s postoperative condition and symptoms in the area of colorectal surgery. Apps that fall within the same category share many similar functionalities, with minimum variance in functionality. It is fair to state that apps that fall into different categories are not mutually exclusive in their functionalities regarding their category inclusion. Across all app categories, studies have indicated a potential benefit of apps, except for the categories of communication and prognosis. Users of apps generally seemed to be satisfied with the apps, while reported patient engagement was highly variable across the categories and domains. Patient engagement with the app is, of course, a driver of the potential clinical effect of apps aimed at patient care. Patient engagement not only depends on the specific features that the app offers but also relates to the context and phase of care the patient is receiving, the patients’ digital literacy, and the apps’ overall usability and stability. Most studies did not report participants’ digital literacy, although it can be assumed that participants had sufficient proficiency, as patients with insufficient proficiency probably did not participate. It is important to acknowledge digital literacy and to compensate for digital literacy as well as possible, as the effectiveness of apps may be substantially less.

Although over 150 gastrointestinal surgical apps for use on a smartphone or tablet are available in the app stores, only a limited amount (29) is reflected in studies as could be retrieved from scientific literature by this systematic review [54–56] Non-validated or poorly validated apps are potentially harmful, especially if they may have a direct effect on clinical outcomes such as diagnosis or decision support tools. This underlines the need for high quality clinical research to safeguard the effectiveness and safety of apps, and to provide HCP's a better understanding of the potential impact of an app on surgical care. It is important to realize that apps can be published in the app stores claiming to be effective or reliable without presenting a snippet of evidence to support clinical safety or efficacy. There are no specific rules or regulations in the submission guidelines for the app stores, which is an important issue [57, 58]. When scientific evidence is needed to safeguard the efficacy, quality and safety of apps to be in clinical settings, and with the medical device regulations in place, the public should at least be able to discern apps that are built and proofed reliably from those that are not before they are downloaded and granted permission from the user. App stores are encouraged to change their submission guidelines for apps that act as a medical device.

Healthcare apps which are used to monitor, guide, diagnose, or treat patients must be regarded as a medical device and thereby have to comply to medical device regulations (FDA or MDR).[8, 9]. The regulations have strict requirements for the (technical) development, validation and quality surveillance of the app, and the manufacture itself. Even with legislation in place, HCP’s or manufacturers may be unaware of the importance of such legislation, which may impede the quality and safety of apps. Although apps evaluated in a clinical study do not have to fully comply to the regulations, it is worthwhile to note that only one author has mentioned the regulations [39]. It is unclear if other apps would be allowed under the medical device regulations. However, it is not guaranteed that the app will lead to valid outcomes if they have met the regulations [7, 10]. Therefore, well-designed scientific research validating apps are needed. As with researching medical devices or drugs, conducting research with healthcare apps is time- and cost-consuming. The role of app manufacturers with commercial interests and eagerness of the public to use apps are potential hazards. It is essential that an expert HCP is involved in the development and validation of healthcare apps. Not only to safeguard content, but also to ensure that apps are well researched and vetted before they become accepted in clinical practice. Although the development process of the apps identified in this review has been rarely or obscurely described, the involvement of HCP is presumed. HCP’s are mostly not involved in unvalidated apps which are available in the app stores, resulting in a potential higher risk [51]. Moreover, apps that collect and/or process medical data must comply with data privacy regulations [59, 60] Specific standards needs to be followed, but not all app manufacturers are familiar with them [61]. Most of the included apps collect or process patient data (25/29), however, only three have mentioned privacy measures [30, 48, 50]. This does not have to imply that these apps do not comply with data privacy regulations as the development process was generally obscurely described.

Since the use of apps in healthcare has grown rapidly, hospitals and health insurers are increasingly demanding that apps are adequately validated before deployment in clinical care. However, they struggle with the minimum required proof of evidence. Conventionally, a RCT is the golden standard, and is especially applicable for high-risk apps which are classified as medical devices. But there are also other methods to validate apps of which mixed methods studies are an excellent example [62]. It is important that all evaluations are published, to shape the proof of evidence of apps. It is recommended that medical apps used in research or clinical practice comply with the suggestions summarised in Table 4.

**Table 4:** Suggestions for future research and/or practice

Process	Suggestions
App development	An ‘expert’ healthcare provider should be involved to safeguard medical content and to ensure that apps are well researched and vetted
	Medical apps should also be compensated for patients with low digital literacy
App evaluation in clinical research	All medical apps should be evaluated on their effectiveness and safety in quality studies in which a control group, objective outcomes on effectiveness of apps and valid and reusable questionnaires are used
	The development process of medical apps should be completely described so that it is possible to assess whether all conditions are met
Regulations in app stores	All medical apps should provide evidence on their effectiveness and safety before the app stores accept their publications
Clinical practice	Healthcare providers and patients must be aware of the level of evidence of apps that they prescribe or use
	Only well-validated medical apps should be used in clinical practice, as high level of evidence is needed to guarantee their efficacy, quality and, safety

## Conclusion

Healthcare providers and patients must be aware of the level of evidence of apps that they prescribe or use. Although apps may offer great potential to improve gastrointestinal surgical care, only a limited number of available gastrointestinal surgical apps have been researched and described in peer-reviewed literature to date. It is of great concern that most studies evaluating gastrointestinal surgical apps fail to generate a high level of scientific evidence, needed to guarantee the efficacy, quality and safety of apps. To fully utilize the potential of gastrointestinal surgical apps in standard surgical care, more and higher quality of research is needed.

## Supplementary Information

Below is the link to the electronic supplementary material.

Supplementary file1 (DOCX 12 KB)
