# Supplementary material for: Mobile applications in gastrointestinal surgery: a systematic review
Source: Surg Endosc. 2023 Apr 4;37(6):4224–48. doi: 10.1007/s00464-023-10007-y (PMC10234873; doi:10.1007/s00464-023-10007-y)
Supplement: Supplementary file 1 — Supplementary file1 (DOCX 12 KB) [file 464_2023_10007_MOESM1_ESM.docx]

**Appendix**

The following key terms and mesh headings were used for the literature search:

("Digestive System Surgical Procedures"[Mesh] OR "Anastomosis, Roux-en-Y"[Mesh] OR "Cholecystostomy"[Mesh] OR "Choledochostomy"[Mesh] OR "Gastric Bypass"[Mesh] OR "Portoenterostomy, Hepatic"[Mesh] OR "Bariatric Surgery"[Mesh] OR “Splenectomy”[Mesh] OR “Colorectal surgery” [Mesh] OR “Sphincterotomy”[Mesh] OR "Roux-en-Y"[tiab] OR “esophagectomy”[tiab] OR “gastrectomy”[tiab] OR "Gastroenterostomy"[tiab] OR “gastro-enterostomy”[tiab] OR "Gastric Bypass"[tiab] OR “Bariatric Surgery"[tiab] OR "Pancreaticojejunostomy"[tiab] OR "Cholecystostomy"[tiab] OR "Choledochostomy"[tiab] OR "Portoenterostomy"[tiab] OR (("Pancreatic*"[tiab] OR "hepatico*"[tiab]) AND “surgery” [tiab]) OR “pancreaticoduodenectomy” [tiab] OR "Jejunoileal Bypass"[tiab] OR “Splenectomy”[tiab] OR “Colorectal surgery” [tiab] OR “Colectomy” [tiab] OR “Hemicolectomy” [tiab] OR “colonic resection” [tiab] OR “rectal surgery” [tiab] OR “TME” [tiab] OR “Total mesorectal excision” [tiab] OR “Ileostomy“ [tiab] OR “Colostomy” [tiab] OR “transanal resection” [tiab] OR “APR” [tiab] OR “abdominoperineal resection” [tiab] OR “Pelvic exenteration” [tiab] OR “Rectopexy” [tiab] OR “J-pouch” [tiab] OR “Sphincterotomy” [tiab])

AND

("Mobile Applications"[Mesh] OR “app”[Tiab] OR “apps”[tiab] OR “mobile application*” [tiab] OR “Mobile device*” [Tiab] OR “Mobile health application*” [Tiab] OR “Smartphone*” [Tiab] OR "mHealth" [tiab] OR (“software” [Tiab] AND  (“Mobile” [Tiab] OR “smartphone” [Tiab]))).
